# Mechanically Exfoliated InP Thin Films for Solar Energy Conversion Devices

**DOI:** 10.1002/smsc.202400167

**Published:** 2024-10-30

**Authors:** Bikesh Gupta, Parul, Yonghwan Lee, Joshua Zheyan Soo, Sonachand Adhikari, Olivier Lee Cheong Lem, Chennupati Jagadish, Hark Hoe Tan, Siva Karuturi

**Affiliations:** ^1^ Department of Electronic Materials Engineering Research School of Physics The Australian National University Canberra ACT 2600 Australia; ^2^ Advanced Batteries Research Center Korea Electronics Technology Institute (KETI) 25, Saenari‐ro Bundang‐gu Seongnam‐si Gyeonggi‐do 13509 Republic of Korea; ^3^ Department of Chemical Engineering Monash University Malaysia, Jalan Lagoon Selatan Bandar Sunway 47500 Selangor Malaysia; ^4^ ARC Centre of Excellence for Transformative Meta‐Optical Systems Research School of Physics The Australian National University Canberra ACT 2600 Australia; ^5^ Australian National Nanofabrication Facility The Australian National University Canberra ACT 2600 Australia; ^6^ School of Engineering The Australian National University Canberra ACT 2600 Australia

**Keywords:** InP, photoanode, solar cell, spalling, thin film

## Abstract

III‐V semiconductors are favoured photo absorber materials for solar energy conversion due to their ideal bandgap, yet their high‐cost hinders widespread adoption. Utilizing thin films of these semiconductors presents a viable way to address the cost‐related challenges. Here, a novel mechanical exfoliation technique is demonstrated, also known as controlled spalling, as a cost‐effective and facile way to obtain thin films of III‐V semiconductors. As a proof of concept, 15 μm thick InP films are successfully exfoliated from their original wafers. Thorough characterization using cathodoluminescence and photoluminescence spectroscopy confirms that the opto‐electronic properties of the exfoliated InP films remain unaffected. Utilizing these InP thin films, InP thin‐film heterojunction solar cells with efficiencies exceeding 13% are demonstrated. Additionally, InP photoanodes are fabricated by integrating NiFeOOH catalyst onto these InP thin‐film solar cells, achieving an impressive photocurrent density of 19.3 mA cm^−2^ at 1.23 V versus reversible hydrogen electrode, along with an applied bias photon‐to‐current efficiency of ≈4%. Overall, this study showcases the efficacy of controlled spalling in advancing economically viable and efficient III‐V semiconductor‐based solar energy conversion devices.

## Graphical abstract

This manuscript presents a significant demonstration in the field III‐V semiconductor solar energy conversion devices, focusing the potential of thin film III‐V semiconductors in conjugation with heterojunction solar cell architecture in addressing the cost‐related challenges. We introduce a novel mechanical exfoliation technique known as controlled spalling, which offers a cost‐effective and facile approach to obtaining thin films of III‐V semiconductors.

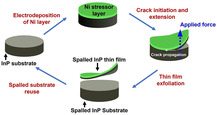

## 1 Introduction

The increasing concerns over climate change and the depletion of fossil fuel reserves have prompted a global shift toward exploring renewable energy sources to meet global energy demands.^[^ ^1^ ^]^ Among several renewable energy sources, solar energy harvesting stands out as a promising solution as the earth receives nearly 9600 times larger energy than today's global energy consumption (17.91 TW in 2017).^[^ ^2^ ^]^ Though solar energy is surplus to meet all our energy demands, the challenge lies in the development of economically and practically viable devices for its efficient harvesting and conversion.

III‐V semiconductors have attracted considerable attention in the field of solar energy harvesting devices because of their ideal bandgap, which allows for maximum absorption of photons and achieving superior efficiency in converting solar energy.^[^ ^3^ ^]^ Additionally, these semiconductors have superior resistance to radiation‐induced damage, making them suitable for space applications.^[^ ^4^, ^5^ ^]^ Moreover, these semiconductors have enabled the transformation of solar energy into electricity and green hydrogen fuel with efficiencies approaching 30%, a milestone unmatched by any other semiconductor material.^[^ ^6^, ^7^ ^]^ Despite their outstanding performance, the widespread utilization of III‐V semiconductor‐based solar energy conversion devices have been limited by their high manufacturing cost, primarily attributed to the high cost of semiconductor wafers and reliance on expensive epitaxial growth for device fabrication.^[^ ^8^ ^]^ In order to improve the cost‐effectiveness of III‐V semiconductor solar energy devices, it is important to utilize thin film technologies for these semiconductors and explore device architectures that could be epitaxy‐free.^[^ ^8^ ^]^

Epitaxial lift‐off (ELO) process has been extensively explored since late 1970's as a way to obtain III‐V semiconductor thin films.^[^ ^9^ ^]^ The process involves selective etching of a sacrificial layer epitaxially grown between the thin‐film semiconductor layer and the substrate. Although this process has been successful in obtaining III‐V semiconductors thin films, the high surface roughness of the parent substrate as well as reaction residues left on the substrate after the ELO process necessitates post‐processing steps, for example, chemical‐mechanical‐polish in order to re‐use the substrate for next epitaxial growth.^[^ ^10^, ^11^ ^]^ Additionally, the etching of the sacrificial layer is a time intensive procedure and involves the use of hazardous chemicals, necessitating the exploration of alternative methods to overcome these challenges. Controlled spalling process is a recently developed technique which potentially presents remedies for the aforementioned issues.^[^ ^12^ ^]^ In this process, a desired thickness of III‐V semiconductor thin film can be peeled off from its thick donor substrate using mechanical loads induced by a stressor layer on top of the mother substrate.^[^ ^13^ ^]^ The spalling process facilitates the production of multiple thin films, ranging in thickness from a few micrometers to several tens of micrometers, from a single‐donor substrate. This approach offers promising opportunities, allowing for the utilization of only the required thickness and facilitating flexible device development.^[^ ^14^ ^]^

Moreover, heterojunction solar cell architecture utilizing carrier‐selective contacts presents a simplified fabrication process for photovoltaic devices compared to epitaxially grown homojunction counterparts, potentially leading to further reductions in production costs. III‐V semiconductor heterojunction solar cells, employing electron‐selective contacts such as TiO_2_, ZnO, and Ta_2_O_5_, have demonstrated efficiencies nearing 20% with fewer fabrication steps.^[^ ^15^, ^16^, ^17^ ^]^ For instance, Reddy et al. have demonstrated InP heterojunction solar cells utilizing Ta_2_O_5_ electron‐selective contacts with efficiencies of 19.1%.^[^ ^17^ ^]^ Similarly, Yin et al. have reported InP heterojunction solar cells with TiO_2_ electron‐selective contacts with efficiencies of 19.2%.^[^ ^15^ ^]^ Recently, Raj et al. showcased a GaAs solar cell incorporating dual TiO_2_ and ZnO electron‐selective contacts, achieving efficiencies of around 21%, underscoring the potential of carrier‐selective contacts to rival homojunction counterparts in efficiency.^[^ ^18^ ^]^ In spite of this, the heterojunction solar cell exhibits superior performance without requiring elaborate epitaxial growth methods. However, the adoption of directly exfoliated InP thin films in heterojunction‐type solar cells has not yet been reported due to the risk of facile cracking in the ultrathin crystalline InP layer. Utilizing the advantages of heterojunction solar cell architecture with InP thin films, our approach aims to minimize manufacturing complexities and reduce material usage. This strategy enhances the economic feasibility of widespread deployment of InP‐based photovoltaic devices

Furthermore, in addition to converting solar energy into electricity, thin‐film III‐V semiconductor devices have the potential for low‐cost production of green H_2_ through photoelectrochemical (PEC) processes. By employing III‐V semiconductor‐based photoelectrodes, researchers have successfully facilitated solar‐driven water‐splitting reactions and thus offering a sustainable route to green H_2_ production.^[^ ^19^ ^]^ For instance, nanostructured InP photocathodes have demonstrated a half‐cell solar‐to hydrogen efficiency of ≈14%.^[^ ^20^ ^]^ Similarly, Gao et al. have demonstrated buried p/n junction InP photocathodes with half‐cell solar‐to‐hydrogen efficiency of 15.6%.^[^ ^21^ ^]^ While significant advancements have been made in developing InP photocathodes, research focusing on InP photoanodes still remains limited. Additionally, the predominant use of thick InP wafers for manufacturing these photoelectrodes increases the overall cost associated with green H_2_ production. Utilizing thin films of these semiconductors into PEC devices have potential for cost reduction and could expedite the commercialization of green hydrogen technologies.

In this study, we leverage the benefits of heterojunction solar cell architecture in conjunction with semiconductor thin films to propose a cost‐effective approach for InP semiconductor solar energy conversion devices. As a proof of concept, we have successfully exfoliated 15 μm thick, ultra‐smooth InP films using the controlled spalling method. Optical characterizations have shown that the radiative recombination of exfoliated InP films remains unaltered. Leveraging these InP thin films, we have successfully developed flexible InP thin‐film heterojunction solar cells with efficiencies surpassing 13%. Furthermore, through the integration of NiFeOOH catalyst onto these InP thin‐film solar cells, we have achieved remarkable results, including a photocurrent density of 19.3 mA cm^−2^ at 1.23 V versus reversible hydrogen electrode (RHE), and an applied bias photon‐to‐current efficiency of around 4%. This comprehensive study underscores the effectiveness of controlled spalling in advancing economically viable and efficient III‐V semiconductor‐based solar energy conversion devices.

## 2 Results and Discussion

### 2.1 InP Thin‐Film Fabrication and Characterization

**Figure 1:**
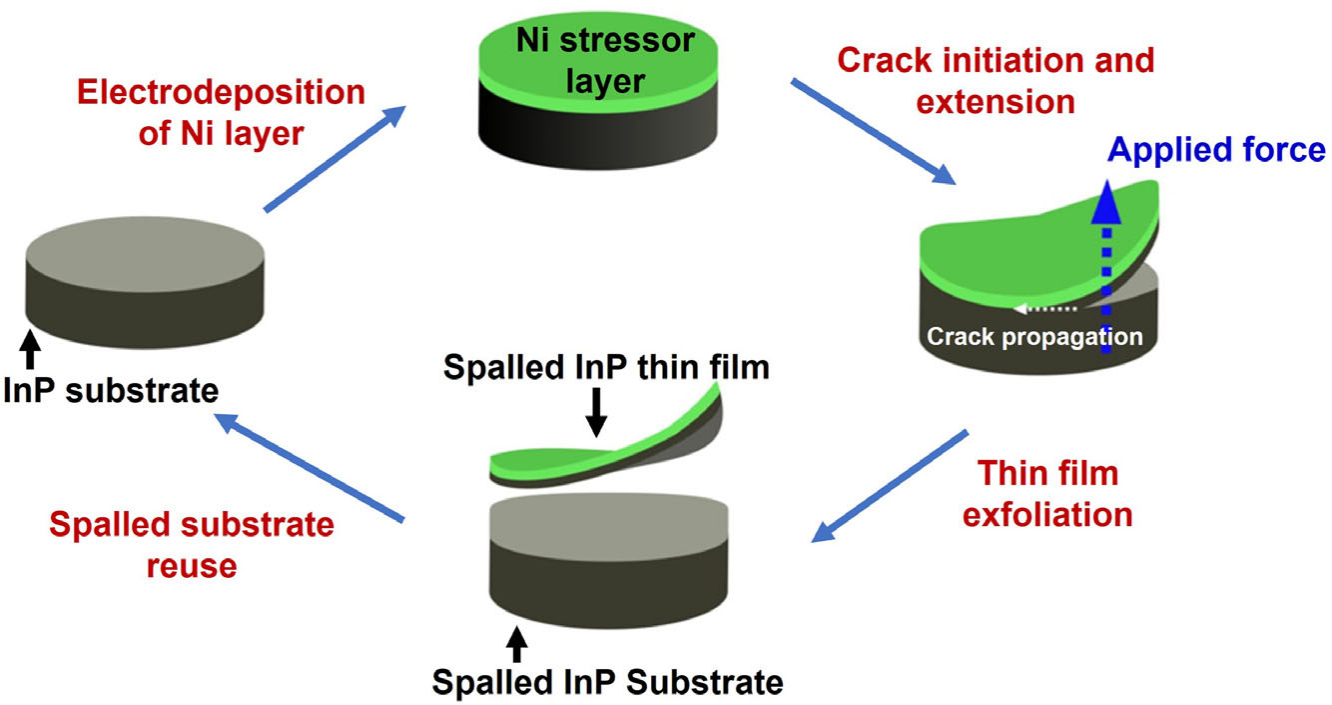
Schematic representation of the overall controlled spalling process.

**Figure** **1** depicts the overall spalling process of (110) p‐type InP substrates, which involves electrodeposition of the Ni stressor layer, initial crack generation and then exfoliating an InP thin film from the donor substrate (for detailed procedure, refer to the experimental section).^[^ ^22^ ^]^ In the spalling process, the initial sub‐surface crack and its specific depth are determined by the thickness and Young's modulus of both the Ni stressor layer and the InP donor substrate.^[^ ^23^ ^]^ The initial sub‐surface crack can be horizontally extended by applying an external force, as indicated by the blue dotted arrow in Figure 1, to separate the thin films from the InP donor substrate.

**Figure 2:**
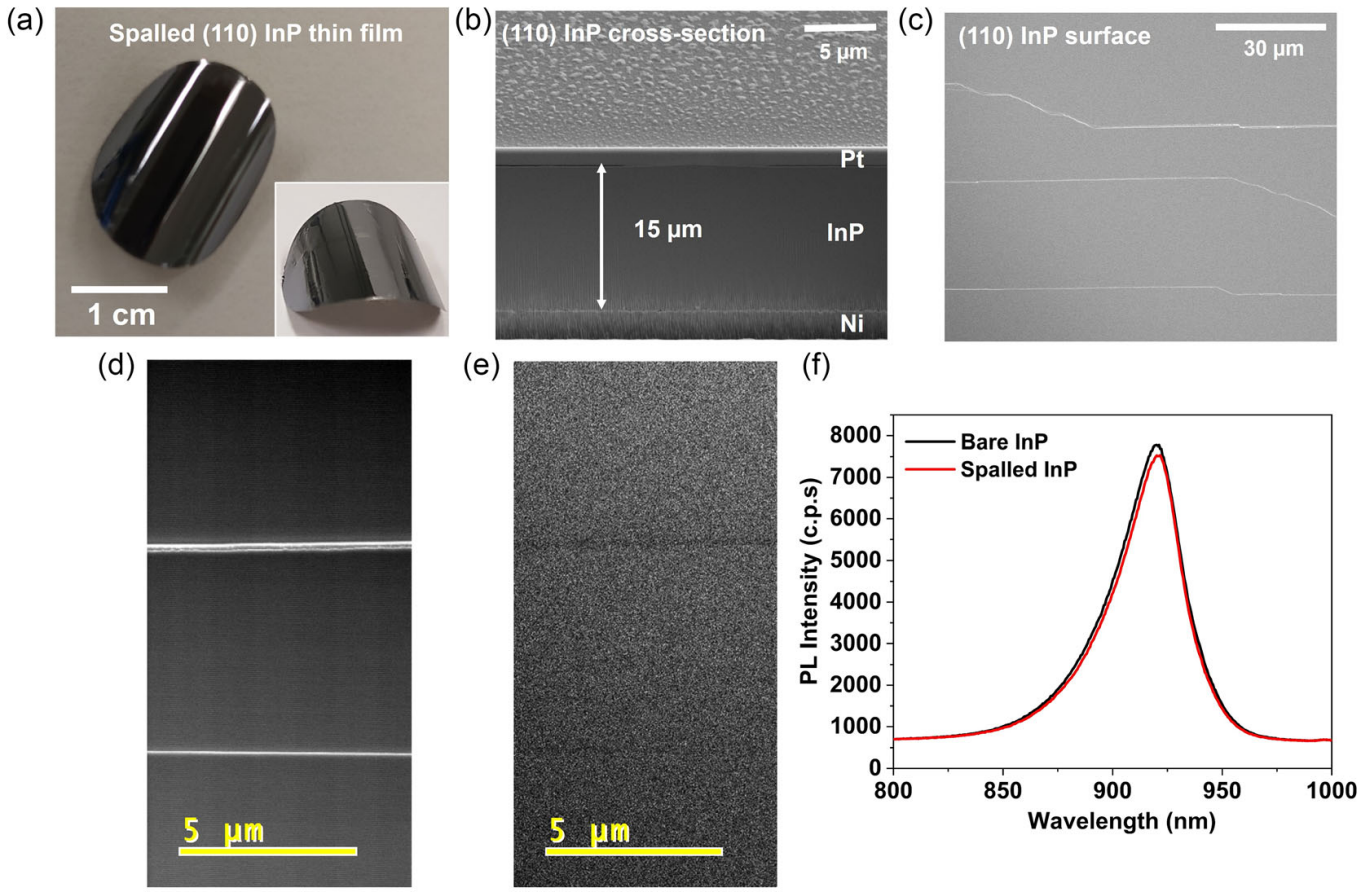
a) Optical photograph of the 15 μm thick InP film. The curvature in the film is due to stress generated by the Ni stressor layer. The inset shows the tilted view of the InP film. b) Cross‐sectional and c) top surface scanning electron micrograph views of the exfoliated InP (110) film while spalling was executed along <110> direction. d) Scanning electron micrograph and e) its corresponding CL spectroscopy micrograph of the exfoliated film. f) PL spectra of bare InP (substrate) and exfoliated InP film.

In **Figure** **2**a, the optical image of a 2 cm diameter exfoliated InP thin film is depicted, with the electrodeposited Ni stressor layer on the rear side. The exfoliated InP thin film exhibits an outward curvature owing to the tensile stress induced by the Ni stressor layer.^[^ ^24^ ^]^ Prior to the electrodeposition of the Ni stressor layer, a thin layer of Zn/Au was deposited with the aim of establishing a rear Ohmic contact to the p‐type InP for solar energy harvesting devices. Additionally, the Ni stressor layer serves dual purpose as an electrical contact layer and as a handling layer, thus preventing the breakage of the exfoliated thin films. Figure 2b displays the cross‐sectional scanning electron microscopy (SEM) micrograph of the exfoliated InP thin film. As observed from the micrograph, the thickness of the InP thin film is 15 μm, excluding the thickness of the Ni stressor layer. The exfoliated InP thin film is 20 times thinner than that of the donor substrate, demonstrating the effectiveness of the spalling process in obtaining thin films without kerf loss.

Furthermore, this thickness of InP is adequate for complete absorption of solar irradiation, owing to the direct bandgap and high absorption coefficient of InP.^[^ ^25^ ^]^ Figure 2c displays a top‐view SEM micrograph of the exfoliated InP film, showcasing a smooth fractured surface. The presence of a smooth fractured surface is characteristic of zinc‐blende InP oriented along the (110) planes unlike (100) oriented zinc‐blende InP where triangular grating structures are formed (Figure S1, Supporting Information). These (110) planes are recognized as the preferred cleavage planes for zinc‐blende InP, facilitating the formation of smooth fracture surfaces.^[^ ^14^ ^]^ Moreover, the smooth fractured surface of the exfoliated (110) film can be highly beneficial for epitaxial growth or wafer‐bonding processes, such as integrating III–V materials with Si.^[^ ^26^ ^]^ Additionally, line‐like surface structures are visible on the InP surface, possibly resulting from uneven application of mechanical force during the spalling process.^[^ ^27^ ^]^ The existence of these surface structures was further verified through atomic force microscopy (Figure S2, Supporting Information). The step height of these structures is around 265 ± 30 nm and has the average surface roughness, Rq, of 225 ± 12 pm. Similar line‐like surface structures were also previously observed in other semiconductor materials such as Si and Ge.^[^ ^27^, ^28^ ^]^

The impact of these surface structures on the optical characteristics of the exfoliated thin InP film is investigated using cathodoluminescence (CL) and photoluminescence (PL) spectroscopy. In Figure 2d,e, both the SEM and corresponding CL micrographs are presented. It is noted that the CL emission intensity diminishes in the regions containing line‐like surface structures, indicating potential non‐radiative recombination centres.^[^ ^29^ ^]^ Despite the nominal decrease in CL intensity in these areas, the impact on the optical properties appears minimal. This is supported by the almost identical PL emission observed in both the reference (substrate) and exfoliated film (Figure 2f), indicating an identical energy bandgap (≈1.34 eV at room temperature) with InP substrate. However, it is still advantageous to minimize surface structures on the exfoliated films. This could potentially be achieved by employing an automated process to ensure uniform application of mechanical force during the spalling process.^[^ ^12^ ^]^

### 2.2 InP Thin‐Film Heterojunction Solar Cell

We utilized the exfoliated InP thin film to fabricate lightweight InP heterojunction solar cells. Our approach employed InP thin films (15 μm) as the light absorption layer. To assess the optical absorption performance as a function of InP thickness, we conducted optical simulations to analyze the achievable photo‐generated current density depending on InP thickness. Figure S3, Supporting Information, exhibits the calculated photo‐generated current density and absorption spectra depending on the InP thickness. The results indicate that the InP thickness has an insignificant effect on the photo‐generated current density depending on InP thickness. For example, when the InP thickness was dramatically reduced from 500 to 15 μm, the change in photo‐generated current density was only ≈3.17%. Due to the high optical absorption coefficient and direct‐band gap of InP, both the spalled thickness (15 μm) as well as a thickness of only a few micrometers are sufficient to absorb solar radiation.^[^ ^30^ ^]^ We also conducted simulations of the photo‐generated current density and absorption spectra for the InP/Ni and ITO/TiO_2_/InP/Ni device structures (Figure S3a,c, Supporting Information). The results indicated that the device structure has a more dominant effect on the photo‐generated current density than the thickness of the InP layer.

III‐V semiconductor heterojunction solar cells based on carrier‐selective contacts is an emerging architecture offering a simplified fabrication process as compared to the traditional homojunction counterparts. Utilizing the heterojunction architecture with thin‐film InP further enhances the cost‐effectiveness of the devices. In our study, InP heterojunction solar cells were fabricated employing TiO_2_ electron‐selective contact (ESC), as the wafer‐based InP heterojunction solar cells with TiO_2_ ESC layer have demonstrated efficiencies approaching 20%.^[^ ^15^ ^]^ However, the fabrication procedures typically involve vacuum‐based thermal processes (*e.g.*, ALD). For thin‐type semiconductor device fabrication, these thermal processes pose significant challenges due to increased vulnerability to external mechanical shock compared to thick semiconductor substrates (>300 μm), making it difficult to adopt the procedure for thin‐type semiconductors.^[^ ^31^ ^]^ Additionally, we directly exfoliated a 15 μm thick InP layer via a tensile stressed electroplated Ni film, resulting in the spalled InP with a stressor layer exhibiting a bent status (see inset of Figure 2a). While the Ni stressor layer is highly useful for preventing breakage, the undesired warpage poses a significant challenge for adoption in conventional vacuum‐based semiconductor fabrication processes. To overcome these challenges, the development of an appropriate handling method is crucial to ensure the successful transformation of thin‐type semiconductors. To address this issue, we implemented a thick Ni foil and Ag paste bonding method. The Ni foil bonding approach not only flattens the spalled InP thin films but also maintains the top‐bottom device structure. These proposed approaches streamline the integration of conventional heterojunction solar cell fabrication procedures with the spalled InP layer.

**Figure 3:**
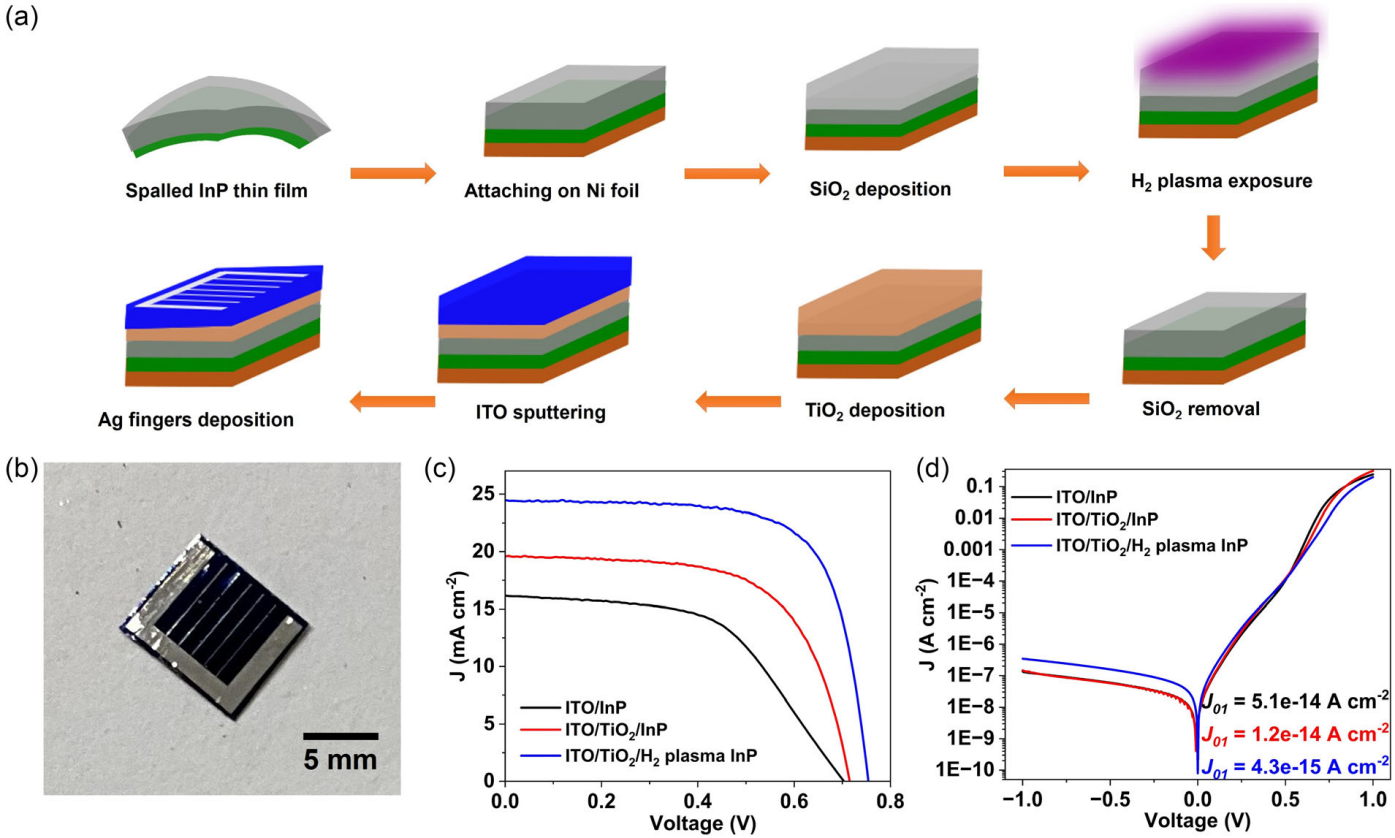
a) Process flow of the InP heterojunction solar cell fabrication. b) Optical image of an InP thin‐film solar cell. c) The light and d) dark *J–V* curves of the fabricated devices.

**Figure** **3**a outlines the overall device fabrication process. In brief, fabrication began by affixing the exfoliated InP film onto a Ni foil using conductive Ag paste, followed by deposition of TiO_2_, ITO, and Ag bus bars. Figure 3b displays the photograph of the fabricated device. By introducing the Ni foil on the bottom side of the spalled InP thin film, the bent film (see Figure 2a) was flattened, facilitating conventional semiconductor fabrication processes such as atomic layer deposition (ALD), sputter, inductively coupled plasma (ICP), and e‐beam evaporation. Figure 3c depicts the light current density–voltage (*J–V*) characteristics of the thin‐film solar cell evaluated under simulated 1 sun irradiation. The reference cell without any ESC layer (ITO/InP) cells demonstrated an open‐circuit voltage (*V*_oc_) of 703 mV, a short‐circuit current density (*J*_sc_) of 16.1 mA cm^−2^, and a fill factor (FF) of 53.9, resulting in an efficiency of around 6.1% (**Table** **1**). However, after insertion of a 10 nm thick TiO_2_ ESC layer, the (ITO/TiO_2_/InP) solar cell exhibited a *V*_oc_ of 714 mV, *J*_sc_ of 19.6 mA cm^−2^, and FF of 63.6, resulting in an efficiency of around 9% (Table 1). There is still room for improvement in the photovoltaic performance of these thin‐film InP heterojunction solar cells. Previous research has indicated that subjecting the InP surface to H_2_ plasma can have a beneficial effect on enhancing the photovoltaic performance of InP heterojunction solar cells.^[^ ^25^, ^30^ ^]^ In line with this, we exposed the InP surface to H_2_ plasma, and as evident from the *J–V* curve, there is an overall improvement in the device performance. The *V*_oc_ increases to 753 mV, *J*_sc_ to 24.4 mA cm^−2^, and FF improves to 70.7, resulting in an efficiency of 13%. It is noteworthy to mention that, the efficiency achieved in the present work is comparable to that of other reported InP thin‐film devices, underscoring the potential of the current architecture to attain high performance while streamlining the fabrication process (**Table** **2**).^[^ ^25^, ^32^, ^33^, ^34^ ^]^ Notably, our *V*_oc_ exceeds by at least 50 mV compared to all previously reported InP thin‐film solar cells highlighting the superior optoelectronic quality of our exfoliated InP thin films. It is to note that the FF in our cells are low probably due to poor adhesion during the affixing of the exfoliated InP film onto a Ni foil using conductive Ag paste.

**Table 1:** Summary of key photovoltaic parameters of the thin‐film InP solar cells.

Device	*V*_oc_ [mV]	*J*_sc_ [mA cm^−2^]	FF [%]	*η* [%]
ITO/InP	703	16.10	53.9	6.1
ITO/TiO_2_/InP	714	19.62	63.6	8.9
ITO/TiO_2_/H_2_ plasma InP	753	24.4	70.7	13.0

**Table 2:** Comparison of key photovoltaic parameters of various InP thin‐film solar cell.

Structure	Thin film process	Device area	Film thickness	*V*_oc_ [mV]	*J*_sc_ [mA cm^−2^]	FF [%]	*η* [%]	References
ITO/InP	Epitaxial lift‐off	0.785 mm^2^	3 μm	620	29.6	55	10.2	[32]
ITO/InP	Epitaxial lift‐off	NA	3 μm	700	31.8	64.4	14.4	[33]
ITO/TiO_2_/InP	Non‐epitaxial growth	1 mm^2^	6 μm	692	26.9	65	12.1	[25]
ITO/TiO_2_/InP	Non‐epitaxial growth	0.25 mm^2^	6 μm	635	26.7	64	10.8	[34]
ITO/TiO_2_/H_2_ plasma InP	Spalling	0.25 cm^2^	15 μm	753	24.4	70.7	13.0	This work

The dark *J–V* characteristics of a solar cell serve as a crucial diagnostic tool for extracting essential information such as recombination current, ideality factor, and cell resistance. Figure 3d presents the dark *J–V* measurements of the InP solar cells, which were fitted using the double‐diode model algorithm.^[^ ^35^ ^]^ The equation used for the dark *J–V* fitting is provided below

(1) $$J=\;J_{01}\exp\frac{\left[q\left(V-JR_{s}\right)\right]}{n_{1}kT}+\;J_{02}\exp\frac{\left[q\left(V-JR_{s}\right)\right]}{n_{2}kT}+\;\frac{V-JR_{s}}{R_{\text{Sh}}}$$

where the parameters *J*_01_ describes recombination current density in the quasi‐neutral bulk region, *J*_02_ denotes Shockley–Read–Hall (SRH) recombination current density in the space charge regions, *n*_1_ and *n*_2_ are the ideality factor of diodes, and Rs and Rsh denote the series and shunt resistances, respectively. The extracted *J*_01_ component of dark current is shown in the inset of Figure 3d.

The *J*_01_ of the ITO/InP reference cell was 5.1 × 10^−14^ A cm^−2^, which reduced to 1.2 × 10^−14^ A cm^−2^ after the addition of the TiO_2_ ESC layer. This indicates a lower recombination rate of photogenerated carriers in InP in the presence of TiO_2_. Furthermore, the *J*_01_ value of the H_2_ plasma‐treated TiO_2_/InP cell reduced by an order of magnitude to 4.3 × 10^−15^ A cm^−2^ compared to the TiO_2_/InP cell. This reduction may be attributed to a lower carrier concentration in the surface region of InP following H_2_ plasma treatment. Additionally, a lower *J*_01_ value is associated with a higher *V*_oc_, as explained by the equation below^[^ ^36^ ^]^

(2) $$V_{\text{oc}}\approx \frac{\;nkT}{q}\text{ ln}\left(\frac{J_{\text{sc}}}{J_{01}}\right)$$

This clearly explains the reason for TiO_2_/InP (*J*_01_ ≈ 1.2 × 10^−14^ A cm^−2^) and TiO_2_/H_2_ plasma‐treated InP (*J*_01_ ≈ 4.3 × 10^−15^ A cm^−2^) cells having higher *V*_oc_ as compared to ITO/InP (*J*_01_ ≈ 5.1 × 10^−14^ A cm^−2^) cells.

The enhancement in photovoltaic performance (*V*_oc_ and *J*_sc_) following H_2_ plasma treatment may be ascribed to a decrease in the carrier concentration within the bulk of InP and carrier type conversion on the surface of InP, as previously observed.^[^ ^15^, ^30^, ^34^, ^37^, ^38^ ^]^ In our previous investigation on the impact of H_2_ plasma treatment on InP, we observed the formation of an n‐type and reduced carrier concentration region in Zn‐doped p‐type InP substrates following H_2_ plasma exposure. The formation of an n‐type region induced energy band bending near the surface, resulting in a built‐in electric field. The combined effect of the energy band bending and the reduced carrier concentration in InP significantly suppressed radiative recombination of photogenerated minority carriers, thereby enhancing both carrier lifetime and diffusion length. Consequently, we noted an improvement in the external quantum efficiency (EQE) and overall efficiency in solar cells utilizing thick (350 μm) wafer‐type InP absorbers. Furthermore, we believe that the H_2_ plasma treatment is similarly effective on a spalled 15 μm thick InP thin film, leading to enhancements in both the *J*_sc_ and *V*_oc_.

Although our results show the highest *V*_oc_ and superior performance compared to other InP thin‐film type solar cells, the efficiency is lower compared to wafer‐type solar cells with identical device structure.^[^ ^37^ ^]^ Specifically, the *J*_sc_ exhibited the most degraded performance parameter compared to the thicker one (Δ*J*_sc_ ≈ 20%). However, as we mentioned, the calculated photo‐generated current density as a function of InP thickness shows no significant effects due to the InP thickness. We anticipate that the degradation originates from the non‐uniform temperature distribution under thermal process resulting from significant warpage of the spalled InP attached to Ni foil. While the attached Ni foil facilitates the flattening of the spalled InP thin film at room temperature, elevated temperature conditions, especially during the ALD process which reaches up to 120 °C for TiO_2_ and 300 °C for SiO_2_ layer deposition, exacerbate this issue. Figure S4, Supporting Information, displays the finite element method (FEM) simulation regarding the deformation and warpage of the InP thin film (15 μm)/electroplated Ni (6 μm)/Ag paste (10 μm)/Ni foil (2 mm) at elevated temperature (120 and 300 °C). Due to the difference in coefficient of thermal expansion (CTE) between the layers used, substantial warpage occurs, leading to significant non‐uniform temperature distribution across the InP thin film under thermal process.^[^ ^39^, ^40^ ^]^ As it is known that the TiO_2_ deposition temperature greatly affects solar cell performance.^[^ ^15^ ^]^ Thus, we anticipate that there is room for improvement in InP thin film solar cells by addressing the warpage issue. Additionally, fill‐factor degradation is induced by the complex backside structure comprising InP/ZnAu/electroplated Ni/Ag paste/Ni foil. Optimization of the contact resistivity of the multiple layers can also improve the fill‐factor and solar conversion efficiency.

Wanlass et al. reported a 24.2% efficiency for an InP solar cell featuring a sophisticated doping profile on the InP absorber along with back surface reflectors.^[^ ^41^, ^42^ ^]^ Their findings indicated that the optimized device structure, incorporating back reflectors, could achieve nearly 100% EQE at longer wavelengths (800–900 nm). This suggests that further refining the device structure, particularly with back reflectors, could improve both the EQE and the *J*_sc_ in spalled InP solar cells. Additionally, Chen et al. showed that an Ag back mirror combined with a nanoscale periodic TiO_2_ grating could enhance the photon recycling effect, leading to a *V*_oc_ increase of about 0.1 V due to higher excitation densities.^[^ ^43^ ^]^ This implies that there is still potential for performance improvement in our spalled InP solar cells by optimizing the back surface reflector in conjunction with a thinned absorber layer. The fill factor could also be improved by implementing advanced multi‐layer metallization techniques or forming contact layers near the InP absorber to achieve low resistivity ohmic contacts, thereby boosting the fill factor.^[^ ^44^, ^45^ ^]^ These advancements could enable spalled InP‐based thin‐film solar cells to achieve efficiencies comparable to those of wafer‐based cells, despite the significantly reduced thickness of the InP.

### 2.3 Light Harvesting and Catalyst Decoupled InP Thin‐Film Photoanode

**Figure 4:**
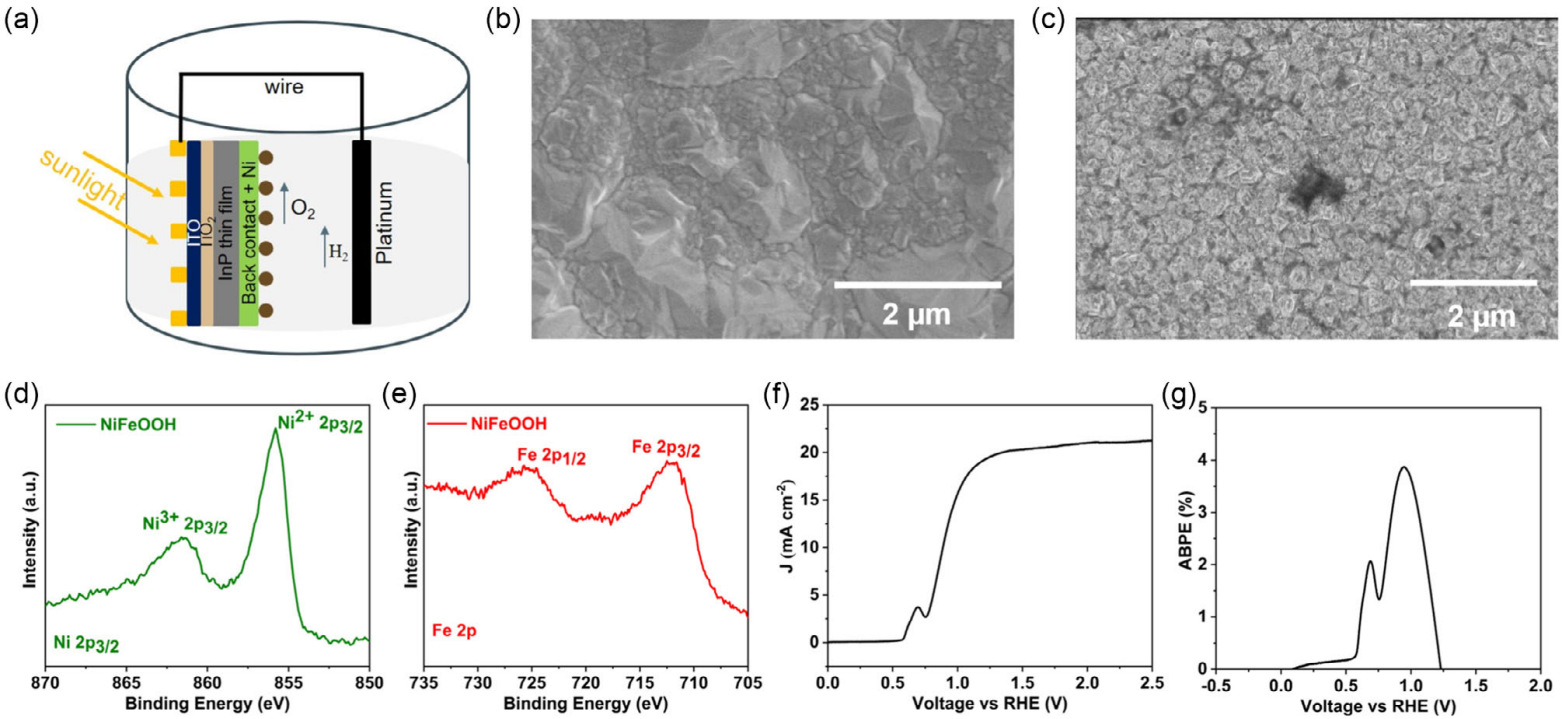
a) Schematic representation of InP photoanode with spatially separated light harvesting and catalytic reaction side. Scanning electron micrograph of the Ni foil b) before and c) after solution corrosion deposition of NiFeOOH catalyst. Core‐level X‐ray photoelectron spectra of d) Ni 2*p* and e) Fe 2*p* of the NiFeOOH catalyst. f) *J–V* curve and g) applied bias photon‐to‐current efficiency of the device in 1.0 M KOH measured under AM1.5G 1 sun illumination.

We further employed the InP thin‐film heterojunction solar cells as photoanodes for the PEC oxygen evolution reaction (OER). Furthermore, we repurpose the Ni foil, initially employed as an electrically conducing handling substrate to flatten the curved spalled InP thin‐film, into an efficient catalytic component by deposition of effective co‐catalyst. Through the incorporation of transparent glass encapsulation on the front side and co‐catalyst coated Ni foil on the rear side, we demonstrated a stable and efficient photoelectrode device structure where light harvesting and catalytic surface are fully decoupled.^[^ ^46^ ^]^ The schematic diagram of the OER facilitated by the InP photoanode is shown in **Figure** **4**a. In the present photoanode configuration, the light‐harvesting side is separated from the oxygen evolution catalytic side. This is unlike the conventional monofacial photoanode configuration where all essential components, such as the light absorber, protective layer, and cocatalysts, are integrated on one side of the device which requires careful selection of different components to ensure they do not compromise light harvesting or catalytic functionality. However, with the current decoupled architecture, it becomes possible to finely tune the front side for optimal light harvesting, while independently optimizing the rear side for the catalytic reaction.^[^ ^47^ ^]^ To facilitate the OER, earth abundant NiFeOOH catalyst was deposited on the rear side (on Ni foil) of the photoanode using solution corrosion method.^[^ ^48^ ^]^ The solution corrosion process has a noticeable effect on the Ni surface. SEM micrograph reveal that after the solution corrosion process, the Ni appears notably rougher (Figure 4c) as compared to pristine Ni (Figure 4b) which is the characteristics typically associated with corroded metals. Additionally, localized darker regions are observed throughout the sample which could be due to Fe‐rich areas.^[^ ^48^ ^]^ Furthermore, analysis of the core‐level XPS spectra indicates the presence of both Ni and Fe in the NiFeOOH catalyst (Figure 4d,e). Moreover, the NiFeOOH catalyst predominantly comprises of oxidized metal species (Ni^2+^, Ni^3+^, and Fe^3+^) (Table S1, Supporting Information), which are known to be highly conducive for OER reactions.^[^ ^49^ ^]^ The PEC performance of the photoanode is assessed and depicted in Figure 4f,g. The light *J–V* curve of the photoanode indicates a saturated photocurrent density of 19.3 mA cm^−2^ at 1.23 V versus RHE. The saturation photocurrent density of the photoanode is observed to be lower than the photovoltaic current density, attributed to reflection losses at the glass interface. Moreover, based on the obtained *J–V* curve, we determined the applied bias photon‐to‐current efficiency (ABPE) to be ≈4% at 0.94 V versus RHE, which is comparatively higher than other InP photoanodes.^[^ ^50^ ^]^

## 3 Conclusions

In summary, we have introduced a novel mechanical exfoliation method, known as controlled spalling, to produce InP thin films. Using this method, we have successfully obtained ultra‐smooth 15 μm thick InP films with optoelectronic properties similar to those of the parent substrate. Importantly, these as‐exfoliated InP thin films can be used straight away for device fabrication, unlike other thin‐film technologies where chemo‐mechanical polishing is necessary. Utilizing these thin films, we have made flexible InP thin‐film heterojunction solar cells employing TiO_2_ as an electron‐selective contact, achieving efficiencies exceeding 13%. Moreover, these thin‐film solar cells were employed as photoanodes for PEC water‐splitting reaction. Notably, this setup spatially decouples the light harvesting and catalytic functionalities. The photoanode demonstrated an ABPE of around 4% and a remarkable photocurrent density of 19.3 mA cm^−2^ at 1.23 V versus RHE, marking a significant advancement in the field. These findings pave the way for the development of scalable and cost‐effective solar energy technologies, contributing significantly to the renewable energy landscape and sustainable future.

## 4 Experimental Section

4.1

4.1.1

##### Materials

Nickel chloride hexahydrate (NiCl_2_·6H_2_O; CAS: 7791‐20‐0), boric acid (H_3_BO_3_; CAS: 7791‐20‐0) and iron chloride (FeCl_3_; CAS: 7705‐08‐0) were purchased from Sigma Aldrich and were used without further purification. Platinum counter electrode and Ag/AgCl reference electrodes were purchased from Gaossunion Co. Ltd.

##### Spalling Process

Mirror‐polished 350 μm thick monocrystalline (110) orientated InP on‐axis wafers (AXT Inc.) doped with Zn (2 × 10^17^ cm^−3^) were utilized as the base substrates. Zn and Au layers, each with thicknesses of 20 and 100 nm, respectively, were sequentially deposited onto the base substrates via DC sputtering (AJA ATC 2400). Subsequently, the substrates underwent annealing in forming gas (5% H_2_, 95% N_2_) at 400 °C for 40 min in a tube furnace. The Zn/Au alloy served a dual role, acting both as a back ohmic contact for the InP devices and as a conductive surface to aid the electrodeposition process. To delineate the electrodeposition area, a circular mask with a diameter of 2 cm was fixed onto the Zn/Au‐coated substrate. Electrodeposition of Ni was conducted in a 0.36 M NiCl_2_ solution bath (21 g of H_3_BO_3_ and 82 g of NiCl_2_.6H_2_O in 600 mL DI water) with a Pt mesh counter electrode, at a current density of 5 mA cm^−2^ to deposit a 6 μm thick Ni stressor layer. Using a mechanical wedge, an initial sub‐surface crack was induced at or near the edge of the stressor layer. This initial crack region was then gripped with tweezer and hand pulled perpendicular to the InP donor substrate. Exfoliation of the InP thin film from the donor substrate occurred once the initial subsurface crack fully propagated to the opposite position of its origin on the donor substrate along <110> direction. Mirror‐polished 350 μm thick monocrystalline (100) orientated InP on‐axis wafers (AXT Inc.) doped with Zn (2 × 10^17^ cm^−3^) were spalled following the same procedure as used for 110 wafers.

##### Material Characterization

Surface morphology of the spalled films and NiFeOOH catalyst was examined using a field emission scanning electron microscope (FESEM; FEI Verios 460) with voltage and current settings of 5 kV and 13 pA, respectively. An atomic force microscope (Bruker) equipped with a Si tip (spring constant 1–5 N m^−1^, resonant frequency 60–100 kHz) was employed to measure surface topography and roughness of the fractured surface of the spalled (110) InP substrate. Photoluminescence spectroscopy was conducted at room temperature using a 522 nm laser source. Cathodoluminescence spectra were captured using a Gatan MonoCL4 system integrated into a FEI Verios 460 Scanning Electron Microscope. Core‐level spectra of the NiFeOOH catalyst were analyzed using X‐ray photoelectron spectrometer (Thermo ESCALAB250Xi) equipped with a monochromatic Al Kα X‐ray source (energy 1486.68 eV).

##### Photovoltaic Cell Fabrication and Measurements

The solar cells were fabricated on 15 μm thick InP substrates. Prior to the fabrication procedure, the exfoliated InP films were affixed to Ni foil using conductive Ag paste (Ted Pella Inc.) to flatten the film before device fabrication. The top surface of the InP was treated by etching in a 5% HCl solution for 15 s to eliminate native oxides from the surface before depositing TiO_2_. A 10 nm thick layer of TiO_2_ was deposited as an electron‐selective contact layer via atomic layer deposition (ALD; using Savannah 100) at 120 °C, employing titanium isopropoxide (Ti[OCH(CH_3_)_2_]_4_) and H_2_O as titanium and oxygen precursors, respectively. Subsequently, a 60 nm‐thick layer of transparent conducting oxide (ITO:In_2_O_3_:SnO = 90%:10%) was sputtered onto the TiO_2_ layer at room temperature in an argon atmosphere (20 sccm, 1.5 mTorr) with an RF input power of 60 W. Lastly, Ag finger grids (with a width and pitch of 50 and 500 μm, respectively) were deposited using a mask and e‐beam evaporation (Temescal BJD‐2000). Following fabrication, the edges of the InP devices were cleaved to define an active area of 5 × 5 mm^2^. For the InP cells treated with H_2_ plasma, initially, a 10 nm thick layer of SiO_2_ was deposited on the InP wafer using plasma‐enhanced atomic layer deposition (P‐ALD: PicoSun – Sunale) at 300 °C, followed by exposure to H_2_ plasma in an inductively coupled plasma system (ICP Samco 400iP) for 5 min at 20 W plasma power. After the plasma exposure, the SiO_2_ layer was removed by immersing the samples in 5% HF for 45 s. Subsequently, TiO_2_, ITO, and Ag were deposited to finalize the device fabrication process. The photovoltaic characteristics of the thin‐film InP heterojunction solar cells were evaluated under simulated one sun conditions, with an illumination intensity of 100 mW cm^−2^, using a Newport solar simulator equipped with an AM1.5G filter. The intensity of the solar simulator was calibrated using a commercial Si photodiode.

##### Photoanode Fabrication

The NiFeOOH catalyst was applied onto the rear side of the device on the Ni foil, to separate the light absorption side from the OER side. The deposition of the NiFeOOH catalyst was achieved through a solution corrosion process of the Ni foil. This involved preparing a 15 mM NiFe chloride solution by combining NiCl_2_.6H_2_O and FeCl_3_ powder at a 1:1 molar ratio in deionized water. Subsequently, the substrate was immersed in the NiFe chloride solution for 1 min, followed by rinsing with deionized water and drying at 70 °C for 1 h. To complete the fabrication of the photoanodes, electrical connections were established on the front side of the device using Ag bars and Ag paint. A glass slide was positioned over the front side of the device, and epoxy was utilized to encapsulate the device, ensuring that the front side remained exposed to solar radiation while the rear side remained accessible to the electrolyte.

##### Photoelectrochemical (PEC) Measurements

PEC measurements were conducted in a three‐electrode setup utilizing a CHI potentiostat (CHI660E, CHI Technologies) under simulated AM 1.5G solar irradiation (100 mW cm^−2^, Newport Sol 3A, Class AAA Solar simulator). The counter electrode employed was a Pt foil (1 × 1 cm), while an Ag/AgCl electrode served as the reference electrode, stored in a 3 M KCl solution. For the electrolyte, a 1 M KOH aqueous solution with a pH of 13.6 was utilized after being saturated with Ar gas for 30 min. Linear sweep voltammetry (LSV) measurements were performed at a scan rate of 5 mV s^−1^. The potentials measured against the Ag/AgCl reference electrode were then converted to the RHE scale using the Nernst equation

(3) $$V_{\text{RHE}}=V_{\text{Ag/AgCl}}+0.198+0.059\times {\text{pH}}_{\text{electrolyte}}$$

where $V_{\text{Ag}/\text{AgCl}}\;$is the potential of the working electrode, $V_{\text{Ag}/\text{AgCl}}$ is the standard redox potential of the Ag/AgCl electrode (0.198 V at 25 °C), and ${\text{pH}}_{\text{electrolyte}}$ is the pH of the 1 M KOH solution (13.6).

The applied bias photon to current efficiency (ABPE) of the photoanode was calculated by using the below equation

(4) $$\eta_{\text{ABPE}}=\frac{\left(1.23-E_{\text{RHE}}\right)\left(V\right)\times J\left({\text{mA cm}}^{-2}\right)\times \eta_{F}}{P\left({\text{mW cm}}^{-2}\right)}$$

where $E_{\text{RHE}}$ represents the applied potential relative to the RHE, *J* denotes the photocurrent density at this applied potential, $\eta_{F}$ denotes the Faradaic efficiency, and *P* indicates the incident lamp power density.

##### Optical Simulation

The photo‐generated current density and optical absorption spectra were simulated using wafer ray tracer (WRT) simulation software (Version 1.6.7, PV Lighthouse Pty. Ltd., Australia). The incident sunlight was chosen to be AM1.5G and the calculation were performed in the wavelength range of 200–1200 nm.

##### Warpage Simulation

The two‐dimensional warpage simulation was analyzed using the structural mechanics module of COMSOL Multiphysics. The simulation structure consisted of 15 μm thick InP, 6 μm thick Ni, 10 μm thick Ag, and 2 mm thick Ni. All lateral dimensions were set to 2 cm.

## Conflict of Interest

The authors declare no conflict of interest.

## Author Contributions

**Bikesh Gupta**: Conceptualization (lead); Data curation (lead); Formal analysis (lead); Investigation (lead); Methodology (lead); Writing—original draft (lead). **Parul**: Data curation (supporting); Writing—original draft (equal). **Yonghwan Lee**: Conceptualization (supporting); Funding acquisition (supporting); Supervision (supporting); Validation (supporting); Writing—review & editing (supporting). **Joshua Zheyan Soo**: Data curation (supporting); Writing—review & editing (supporting). **Sonachand Adhikari**: Data curation (supporting); Writing—review & editing (supporting). **Olivier Lee Cheong Lem**: Data curation (supporting); Writing—review & editing (supporting). **Chennupati Jagadish**: Funding acquisition (equal); Supervision (equal); Validation (equal); Writing—review & editing (equal). **Hark Hoe Tan**: Conceptualization (equal); Funding acquisition (equal); Supervision (equal); Validation (equal); Writing—review & editing (equal). **Siva Karuturi**: Conceptualization (lead); Formal analysis (equal); Funding acquisition (lead); Project administration (lead); Resources (lead); Supervision (lead); Validation (lead); Writing—review & editing (lead).

## Supporting information

Supplementary Material

## Data Availability

The data that support the findings of this study are available from the corresponding author upon reasonable request.
