# Supplementary material for: Mechanically Exfoliated InP Thin Films for Solar Energy Conversion Devices
Source: Small Sci. 2024 Oct 30;4(12):2400167. doi: 10.1002/smsc.202400167 (PMC11935179; doi:10.1002/smsc.202400167)
Supplement: Supplementary file 1 — Supplementary Material [file SMSC-4-2400167-s001.pdf]

Mechanically-Exfoliated InP Thin Films for Solar Energy Conversion Devices

Bikesh Gupta[1#], Parul Parul[1#], Yonghwan Lee[2*], Joshua Zheyan Soo[1], Sonachand Adhikari[1,4], Olivier Lee Cheong Lem[1,3], Chennupati Jagadish[1,4], Hark Hoe Tan[1,4], Siva Karuturi[5*]

[1]Department of Electronic Materials Engineering, Research School of Physics, The Australian National University, Canberra, ACT 2600, Australia.

[2]Advanced Batteries Research Center, Korea Electronics Technology Institute (KETI), 25, Saenari-ro, Bundang-gu, Seongnam-si, Gyeonggi-do, 13509 Republic of Korea.

[3]Australian National Nanofabrication Facility, The Australian National University, Canberra, ACT 2600, Australia

[4]ARC Centre of Excellence for Transformative Meta-Optical Systems, Research School of Physics, The Australian National University, Canberra, ACT 2600, Australia.

[5]School of Engineering, The Australian National University, Canberra, ACT 2600, Australia.

*Corresponding email: bethesky27@gmail.com, siva.karuturi@anu.edu.au

[#] These authors have contributed equally.

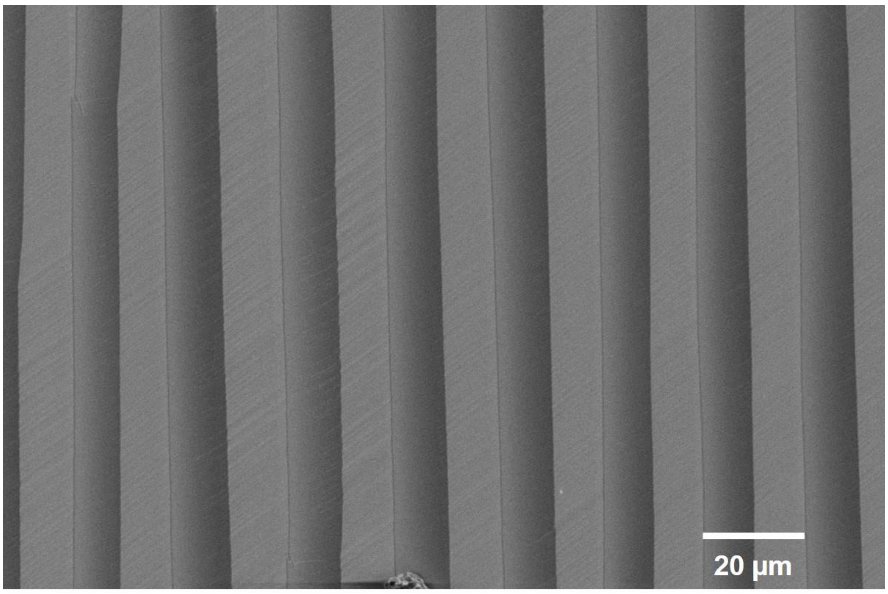

Figure S1. Scanning electron micrograph of an exfoliated InP (100) film.

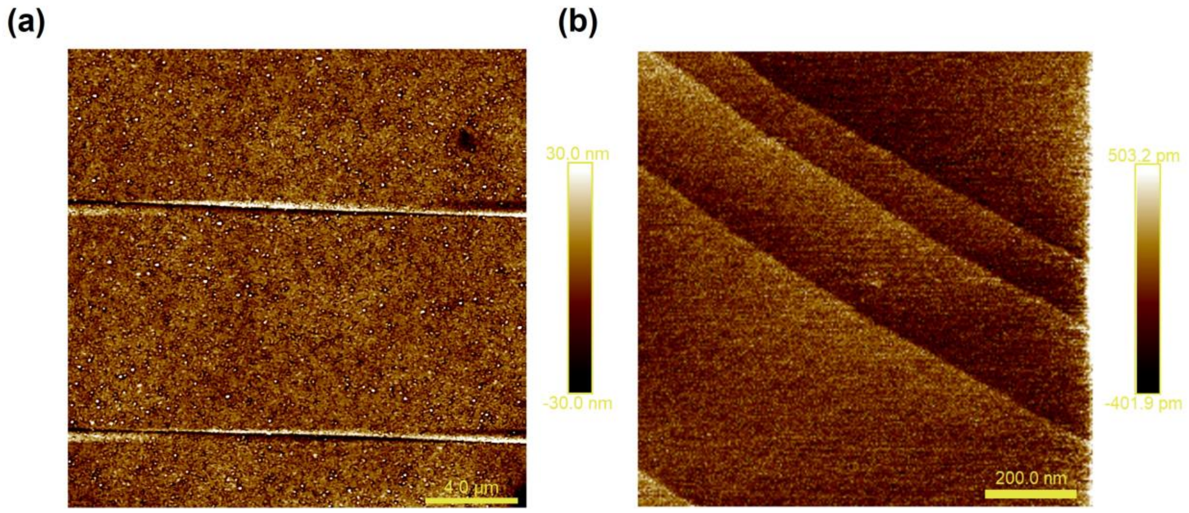

Figure S2. Atomic force micrograph of an exfoliated InP (110) film scanned at different resolution.

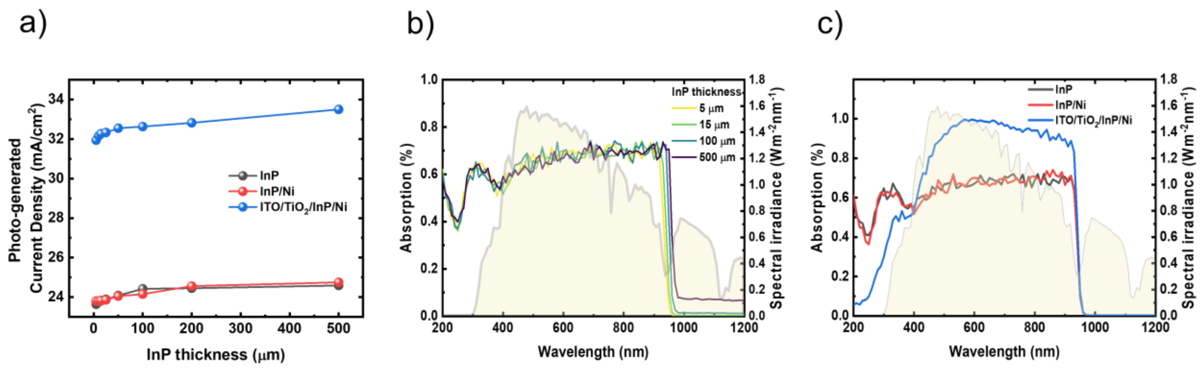

Figure S3. Optical simulation of InP thin film. (a) Simulated photo-generated current density of InP, InP/Ni, and ITO(60 nm)/TiO_2(10 nm)/InP/Ni as a function of InP thickness. Optical absorption spectra of (b) InP as a function of the thickness and (c) solar cell device structure with InP thickness of 15 µm. The photo-generated current density and optical absorption spectra were simulated using Wafer Ray Tracer (WRT) simulation software (Version 1.6.7, PV Lighthouse Pty. Ltd., Australia). The incident sunlight was chosen to be AM1.5G and the calculation were performed in the wavelength range of 200 – 1200 nm.

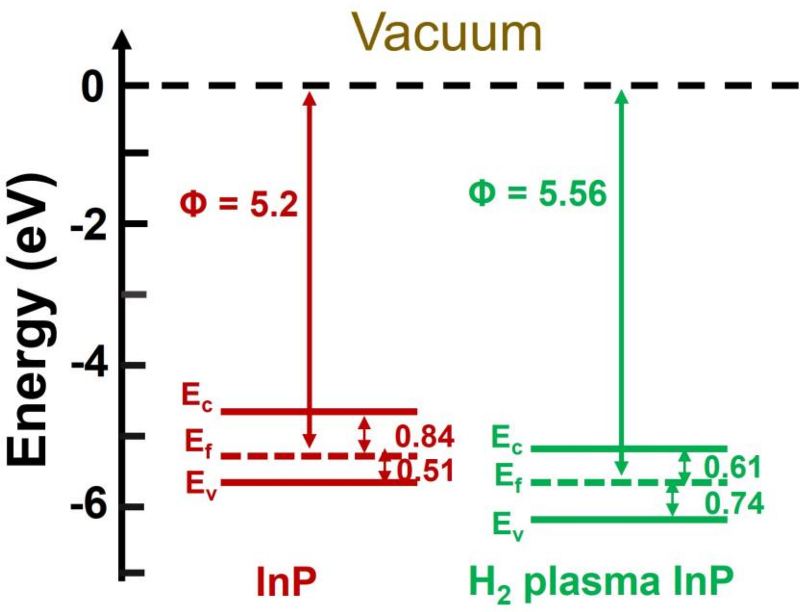

Figure S4. Energy band diagram of InP and H_2 plasma treated InP reflecting the fermi level moving closer to conduction band after H_2 plasma treatment.

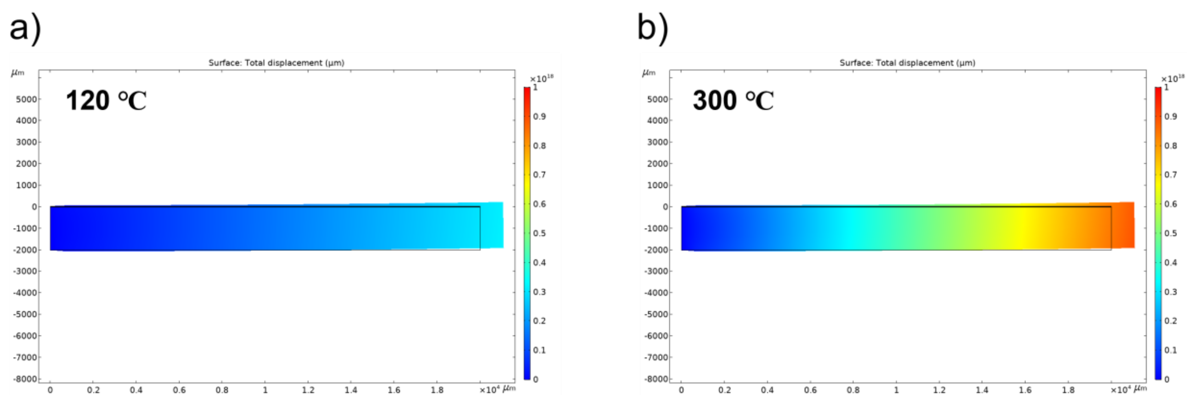

Figure S5. Warpage simulation on the spalled InP thin film attached with thick Ni foil via finite element method (FEA). The total displacement of the InP(15 μm)/Electroplated Ni(6 μm)/Ag paste(10 μm)/Ni foil(2 mm) at (a) 120 °C and (b) 300 °C, corresponding to the atomic layer deposition (ALD) process temperature, corresponding to the ALD process temperature for TiO_2 and SiO_2 layer, respectively.

Table S1. Assignment of Ni 2p and Fe 3p peaks of the XPS spectra in Figure 4 (c) and (d) of the main manuscript.

Ni 2p	Peak	Energy (eV)	Assignment
	1	855.7	Ni^{2+} $2p_{3/2}$
	2	857.5	Ni^{3+} $2p_{3/2}$
Fe 3p	1	55.9 eV	Fe^{3+} 3p
